# Why does leader aggressive humor lead to bystander workplace withdrawal behavior?—Based on the dual path perspective of cognition- affection

**DOI:** 10.3389/fpsyg.2022.925029

**Published:** 2022-09-20

**Authors:** Hao Chen, Liang Wang, Jiaying Bao

**Affiliations:** ^1^School of Economics and Management, Wuhan University, Wuhan, China; ^2^Chinese Graduate School, Panyapiwat Institute of Management, Pak Kret, Thailand; ^3^School of Literature and Media Institute, Baise University, Guangxi, China

**Keywords:** leader aggressive humor, bystander affective rumination, bystander workplace anxiety, bystander workplace withdrawal behavior, bystander organization-based self-esteem

## Abstract

Based on the Cognitive-Affective Personality System Theory, this study takes 443 employees of several Chinese enterprises and their direct superiors as the research objects, then a 1:1 paired survey is carried out at three different time points, and data is processed by Mplus 7.4 software. This study finds from a bystander perspective: leader aggressive humor plays a positive role in bystander affective rumination and bystander workplace anxiety. Both bystander affective rumination and bystander workplace anxiety play a mediation role between leader aggressive humor and bystander workplace withdrawal behavior. Besides, organization-based self-esteem alleviates the positive impact of leader aggressive humor on bystander affective rumination and bystander workplace anxiety, and then moderates the indirect impact of leader aggressive humor on bystander workplace withdrawal behavior through bystander affective rumination and bystander workplace anxiety, respectively. This study has practical guiding significance for promoting the organization to reduce the occurrence of aggressive humor, helping employees better integrate into the organization, and building a harmonious organizational environment.

## Introduction

With the in-depth study of positive psychology, scholars have paid more attention to the impact of a relaxed and pleasant work atmosphere on employees' work performance, and have introduced humor as a variable into the field of organizational behavior research. In the face of the accelerating pace and the increasing pressure of work, humor as a management tool is increasingly sought after and recognized by leaders in the organization. Leader humor is a communication behavior in that leaders consciously amuse a specific subordinate or team through verbal or non-verbal activities (Pundt and Venz, 2017). Leader humor has been widely concerned by scholars because of its positive results, such as improving employees' organizational commitment, job satisfaction, organizational citizenship behavior, and job performance (Vecchio et al., 2009; Robert et al., 2016; Cooper et al., 2018). However, with the deepening of research, the negative side of leadership humor has been gradually exposed, and scholars realize that not all humor is beneficial. Compared with leader positive humor behavior (such as leader affinity humor), leader aggressive humor has gradually attracted scholars' attention in recent years (Wisse and Rietzschel, 2014). Leader aggressive humor refers to the behavior that leaders ridicule, deriding, criticizing, or teasing employees in the form of humor (Martin et al., 2003; Huo et al., 2012), which not only has an impact on the affective reaction and behavior of the ridiculed employees (Huo et al., 2012; Yam et al., 2018) but also destroys the harmonious internal atmosphere of the organization, suppresses the organization's performance, and brings huge economic losses to the organization (Anderson and Ditunnariello, 2016).

Compared with other negative leader behaviors in the workplace, such as abuse management, workplace bullying, and workplace exclusion, the current research on the impact of leader aggressive humor is still focused on the perspective of the taunted person and very few studies on the perspective of bystanders. We have found that the previous studies on the impact of negative workplace leader behaviors on bystander behaviors are not completely consistent. When bystanders see or feel negative events in the workplace, they would show different behaviors because of their different affective reactions (Mitchell et al., 2015; Priesemuth and Schminke, 2019). Moreover, the impact of negative leadership behavior on bystanders can be longer and more far-reaching than on employees who are directly injured (Rosenberg et al., 2021). Therefore, how would a leader aggressive humor arouse the bystanders' feelings and reactions? The existing relevant research domains are not enough to fully explain the inner mechanism. Based on the above analysis, this study intends to further explain the role mechanism between leader aggressive humor and bystanders' behaviors, focusing on the colleagues who are mocked.

According to the Cognitive-Affective Personality System Theory, the individual's environment or event could be activated through the individual recognition unit or affective unit, which would affect individual's attitude or behaviors (Mischel and Shoda, 1995). In the daily work, the leader aggressive humor is a negative work event. Through the perception and evaluation of this event, it is very likely to cause their own affective reaction, and the affections produced in the evaluation process would affect their subsequent actions. Therefore, this study focuses on two variables, affective rumination, and workplace anxiety. The former is a continuous negative perception deviation related to work (Querstret and Cropley, 2012), while the latter is a feeling of nervousness and fear of completing a work task (McCarthy et al., 2016). To a certain extent, negative perceptions and affections would lead to the individual's behavior of flinching in the workplace (Tepper et al., 2008; Wang and Yi, 2012; Chi and Liang, 2013), namely, workplace flinching behavior. Therefore, this study discusses the role of both affective rumination and workplace anxiety between leader aggressive humor and bystander workplace withdrawal behavior. In addition, organization-based self-esteem shows an individual's judgment of role in the organization, which reflects the individual's perception of importance in the organization (Pierce et al., 1989).

**Figure 1:**
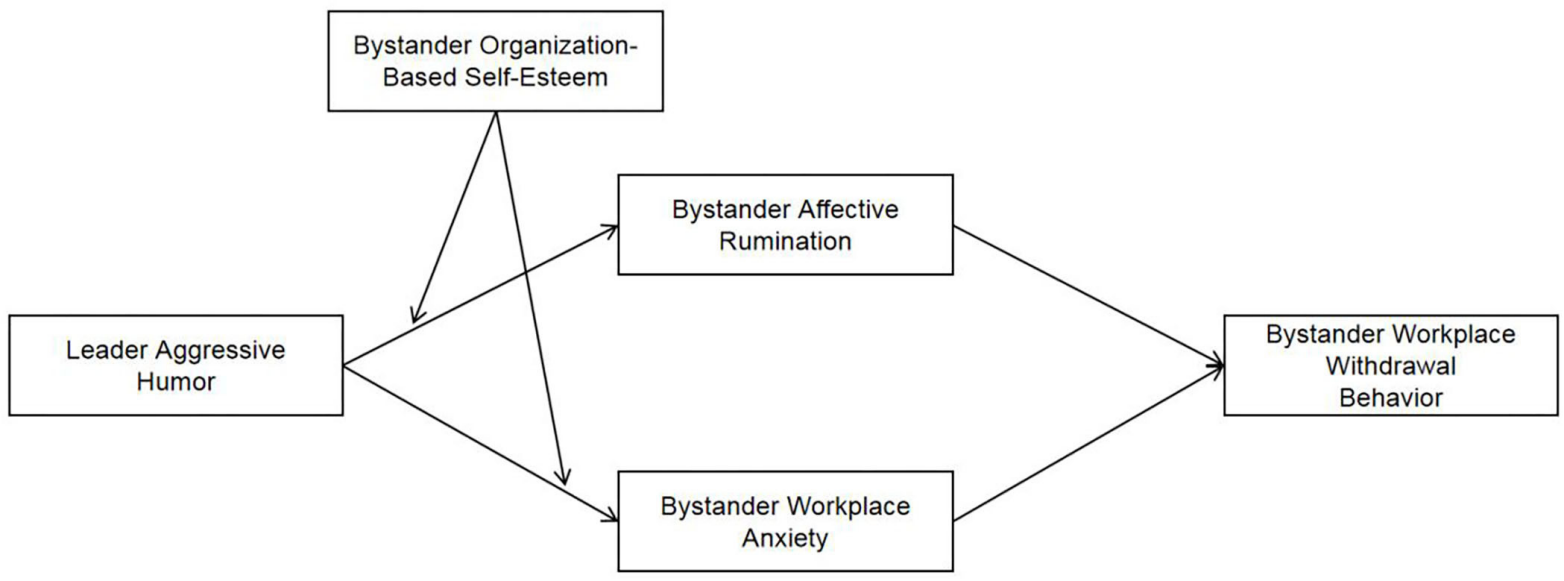
Theoretical hypothesis model.

Based on the above analyses, from the perspective of the bystander, this study takes Cognitive-Affective Personality System Theory as the logic meridian and introduces affective rumination and workplace anxiety as the double medium mechanism. From the two paths, it analyzes the role of leader aggressive humor on bystander workplace withdrawal behavior and also discusses the moderation role of the bystander organization-based self-esteem in the model. This study enriches and expands the impact mechanism of leader aggressive humor, and provides new insights for the study of leader humor. Moreover, this study can also provide relevant guidance to the management practice, such as understanding the possible negative impact of leader humor and reducing the negative impact of leader aggressive humor. The specific research model is shown in Figure 1.

## Literature review and hypotheses

### Leader aggressive humor, bystander affective rumination, and bystander workplace anxiety

Leader aggressive humor is in the form of disrespect, sarcasm, and deliberately making others feel embarrassed or abashed. It is related to teasing, belittling, satire, and slander (Martin et al., 2003). Leader aggressive humor is a direct violation of interpersonal relationships, which cannot be easily remedied by the organization (Huo et al., 2012). In the organization, leader aggressive humor is more destructive than other workplace stress and stimuli, because it takes pleasure in consuming other people's shortcomings or disadvantages (Pundt and Herrmann, 2015), which not only worsens the interpersonal relationship between superiors and subordinates but also cause employees' functional disorder response (Goswami et al., 2015), and induces employees' deviant behavior (Yin et al., 2014). Existing studies have shown that when bystanders witness or perceive other colleagues experiencing negative workplace events, even if they do not suffer the same treatment, they will also be affected anyway (Mitchell et al., 2012; Priesemuth et al., 2014). Therefore, as a bystander, after witnessing or perceiving leader aggressive humor, his cognition and affection may also be affected to a certain extent.

Affective rumination refers to conscious and repeated negative thoughts related to work, which usually occur after work or during leisure time and other non-working hours (Querstret and Cropley, 2012). Research shows that the higher the leaders' job requirements or performance expectations on employees, the more obvious the rumination of employees related to work (Perko et al., 2017; Syrek et al., 2017). This study holds that leader aggressive humor may lead to bystander affective rumination. According to the Cognitive-Affective Personality System Theory, specific events or situational characteristics can stimulate individuals to pay attention to and process external environmental information, so as to form cognitive evaluation and make behavioral decisions (Forgas and George, 2001). As a negative work event, when bystanders witness or perceive that colleagues are ridiculed by leaders, they will process the negative information in the current working environment (Cropanzano and Wright, 2001) to judge whether it is in line with their own values and interests. Obviously, leadership behaviors can violate workplace rules, undermine organizational justice and damage a harmonious working atmosphere (Cooper, 2008), then induce bystanders to have a negative cognitive evaluation of their working environment, resulting in a cognitive units activation and affective rumination. Meanwhile, colleagues who suffer from sarcasm will have problems such as depression, excessive tension, and being out of affection control (Martin et al., 2003; Huo et al., 2012). Additionally, as the communication between colleagues at work is quite frequent, thus, the cognitive evaluation of the ridiculed colleagues may also be transmitted to the bystanders, causing the bystanders to think about how they should get along with the leaders in the future, what they should pay attention to at work, and what will become the targets of the leaders' ridicule, which finally lead to affective rumination. Therefore, the following hypothesis is proposed:

*H1. Leader aggressive humor plays a positive role in bystander affective rumination*.

Workplace anxiety is the feeling that employees feel nervous and worried about completing work tasks (McCarthy et al., 2016), it is the tension and worry that employees feel when they feel potential threats, it represents the stress response of employees with tension as symptoms (Beerhr, 1995). Workplace anxiety is state anxiety in workplace situations, which usually occurs when individuals are facing pressure or specific tasks. Combined with the Cognitive-Affective Personality System Theory, bystanders witness or perceive that leaders show more aggressive humor to colleagues at work, and their affective units may also be activated, which will lead to workplace anxiety. On the one hand, leader aggressive humor is a special form of abusive management (Bamberger and Bacharach, 2006). Bystanders form cognitive evaluations of leader aggressive humor by integrating information in the work environment, which may cause bystanders to worry about their future situation in the organization, question whether the same experience will happen to them, and evaluate the threat, challenge, and harmfulness of the event to themselves. When bystanders perceive that there is a threat to their own goals or interests in the workplace, they often have some negative affective reactions (Beckker et al., 2003; Barlow, 2004). On the other hand, leader aggressive humor behaviors can be regarded as a source of stress in the workplace, and leader aggressive humor words and behaviors can also make bystanders experience negative emotions and make it difficult for them to feel the pleasure of work (Pundt and Herrmann, 2015). Therefore, leader aggressive humor as a threat may activate the affective units of bystanders, thus causing their workplace anxiety. Therefore, the following hypothesis is proposed:

*H2. Leader aggressive humor plays a positive role in bystander workplace anxiety*.

### Mediation role of bystander affective rumination and bystander workplace anxiety

As a negative out-of-role behavior, workplace withdrawal behavior refers to the negative behavior response of employees when dealing with the imbalance between their own pay and organizational return (March and Simon, 1958), it has certain concealment, laziness, avoidance, and retaliation in characteristic, such as being late, leaving early, sleeping at work time, leaving work without reason and not working hard. Research shows that workplace withdrawal behavior is common in organizations, which is not only very unfavorable to employees' career development, but also has a tangible or intangible negative impact on the organization (Viswesvaran, 2002; Zimmerman and Darnold, 2009).

According to Cognitive-Affective Personality System Theory, when facing a specific event or situation, some cognitive units or affective units of individuals will be activated and affect individual behaviors. Therefore, this study holds that leader aggressive humor triggers bystander affective rumination and further stimulates bystander workplace withdrawal behavior. Specifically, when witnessing or perceiving leader aggressive humor, bystanders will form self-cognitive evaluation through information processing. At the same time, the negative impact of leader aggressive humor can lead employees to fall into continuous cognitive bias, reawaken the psychological response of bystanders during non-working hours (Berset et al., 2011), and then activate their cognitive units so that they can't control their thinking (Hobfoll et al., 2018). This negative cognition will further affect the behavior pattern of bystanders, making them tend to take negative coping means such as avoiding work and staying away from the organization, also show work withdrawal behaviors that are not conducive to the development of the organization, such as venting their inner dissatisfaction through negative behaviors like early leave and resignation (Probst, 2002; Podsakoff et al., 2007). Therefore, the following hypothesis is proposed:

*H3. Bystander affective rumination plays a mediation role between leader aggressive humor and bystander workplace withdrawal behavior*.

According to Cognitive-Affective Personality System Theory, leader aggressive humor may lead to employees' workplace anxiety, and then induce their workplace withdrawal behavior. Specifically, leader aggressive humor is a kind of negative humor used by leaders to treat employees, including teasing or ridiculing employees (Cooper, 2008), its essence is to belittle employees under the disguise of playfulness. When leaders show aggressive humor, humor evolves into a source of stress in the workplace (Huo et al., 2012), which will not only make bystanders feel greater psychological pressure but also activate their affective units and produce anxiety experience (Jones et al., 2015). At the same time, a series of negative affective reactions brought by workplace anxiety to bystanders may lead to corresponding negative behaviors (Haines et al., 2002), i.e., stimulating bystanders to alleviate the impact of leader aggressive humor and escape the affective state of anxiety through workplace withdrawal behavior. Besides the unfair and unequal treatment of subordinates by superiors (Cooper, 2008), bystanders will try to fight back (Morrison and Robinson, 1997) by taking negative behaviors, and believing the behaviors that cause harm to leaders or organizations are also appropriate and reasonable. Therefore, the following hypothesis is proposed:

*H4. Bystander workplace anxiety plays a mediation role between leader aggressive humor and bystander workplace withdrawal behavior*.

### Moderation role of bystander organization-based self-esteem

Organization-based self-esteem as a personality trait is individuals' self-cognition and judgment of their importance in the organization, i.e., individuals think they are valuable to the organization and have the ability to create value for the organization (Pierce et al., 1989). Employees with high organization-based self-esteem feel that they are important, influential, and valuable in the organization (Pierce et al., 1989), and their own needs are met through the performance of roles in the organization (Pierce and Garden, 2004), while employees with low organization-based self-esteem think they are unimportant and worthless in their organization. Research shows that employees with high organization-based self-esteem have more positive self-evaluation and subjective efficacy, and can produce more constructive behaviors (Chen and Aryee, 2007).

According to the Cognitive-Affective Personality System Theory, individual traits can explain the relationship between external situations and their cognitive as well as emotional responses (Mischel and Shoda, 1995). Therefore, this study holds that bystander organization-based self-esteem can alleviate the relationship between leader aggressive humor and bystander affective rumination. Specifically, the higher the level of organization-based self-esteem is, the more positive attitude bystanders hold toward themselves and the more confidence they have in themselves, the less they pay attention to others' evaluation of themselves, and the less they are affected by the external situation. When witnessing or perceiving leader aggressive humor toward their colleagues, bystanders with high organization-based self-esteem believe that they have the ability to deal with the pressure brought by leader aggressive humor through their own cognitive evaluation. Bystanders with low organization-based self-esteem are prone to negative evaluation of themselves, they often abandon themselves, doubt their ability level, fear facing challenges, are sensitive to negative information from the outside world, and are vulnerable to negative situations (Yin et al., 2014). Thus, this continuous cognitive response will lead to affective rumination. Therefore, the following hypothesis is proposed:

*H5. Bystander organization-based self-esteem plays a moderation role between leader aggressive humor and bystander affective rumination, that is, the higher the bystander organization-based self-esteem is, the weaker the positive relationship between leader aggressive humor and bystander affective rumination is*.

This study also holds that the strength of the relationship between leader aggressive humor and bystander workplace anxiety is affected by bystander organization-based self-esteem. Specifically, bystanders with high organizational self-esteem usually think that they play an important and meaningful role in the organization (Pierce and Garden, 2004), have a strong sense of identity and responsibility for the organization (Pierce et al., 2016), and tend to regard environmental information in the workplace as opportunities and challenges (Pierce and Garden, 2004). Meanwhile, bystanders with high organization-based self-esteem have strong self-confidence, they can show positive emotional responses at work, they believe that they are competent for their role in the organization (Pierce and Garden, 2004), and they also believe that they will not become the target of leader attack. Bystanders with low organization-based self-esteem are more sensitive to negative organizational situations and lack confidence in themselves, it is easy for them to produce negative work attitudes and behaviors (Bowling et al., 2010), and then aggravate workplace anxiety. Therefore, the following hypothesis is proposed:

*H6. Bystander organization-based self-esteem plays a moderation role between leader aggressive humor and bystander workplace anxiety, i.e., the higher the bystander organization-based self-esteem is, the weaker the positive relationship between leader aggressive humor and bystander workplace anxiety is*.

### Moderated mediation

Based on the above hypotheses, it can be further inferred that the mediation effect of bystander affective rumination and bystander workplace anxiety may be affected by bystander organization-based self-esteem, i.e., leader aggressive humor may lead to bystander affective rumination, bringing the affective state of workplace anxiety, and cause bystander workplace withdrawal behaviors that endanger the development of the organization, such as avoiding working and staying away from work. However, bystander organization-based self-esteem not only reduces the negative effect of leader aggressive humor on bystander affective rumination and workplace anxiety but also reduces the indirect effect of leader aggressive humor on workplace withdrawal behavior through bystander affective rumination and bystander workplace anxiety. Specifically, bystanders with high organization-based self-esteem have a positive evaluation of themselves. They believe that they have the ability to avoid verbal ridicule of leaders and inhibit their affective rumination and workplace anxiety, so it is less likely for them to make workplace withdrawal behavior. Conversely, for bystanders with low organization-based self-esteem, leader aggressive humor has a greater impact on affective rumination and workplace anxiety, and they are more likely to act workplace withdrawal behaviors. Therefore, the following hypotheses are proposed:

*H7. Bystander organization-based self-esteem moderates the mediation role of bystander affective rumination between leader aggressive humor and bystander workplace withdrawal behavior. That is, the higher bystander organization-based self-esteem is, the weaker the mediation role of bystander affective rumination between leader aggressive humor and bystander workplace withdrawal behavior is*.
*H8. Bystander organization-based self-esteem moderates the mediation role of bystander workplace anxiety between leader aggressive humor and bystander workplace withdrawal behavior. That is, the higher bystander organization-based self-esteem is, the weaker the mediation role of bystander workplace anxiety between leader aggressive humor and bystander workplace withdrawal behavior is*.

## Methods

### Participants and procedure

This study takes ordinary employees and their direct superiors of several Chinese enterprises as samples and uses offline questionnaires to collect data. In order to avoid the impact of homology bias, this study conducted a 1:1 employee-direct supervisor matching approach to data collection at three-time points, the time interval for each survey was 1 month. The specific investigation process is as follows: For the first time (T1), the respondents are employees, and the survey includes basic information about employees and leader aggressive humor. For the second time (T2), the respondents are employees, and the survey includes bystander affective rumination, bystander workplace anxiety, and bystander organization-based self-esteem. For the third time (T3), the respondents are employees' direct superiors, and the survey includes bystander workplace withdrawal behavior. Except for some demographic variables, all the questionnaires in this study were scored with a Likert 6-points scale.

In order to enable participants to complete the questionnaire correctly, we have taken the following four measures. First, before distributing the questionnaire, we explained to all participants that the data collected in the questionnaire is only for academic research, not for any other purpose. Second, we promised to pay ¥ 50 (about $7) per person after completing three surveys correctly. Third, in the process of answering the questionnaire, one of our members maintained a close relationship with the participants to solve any problems they raised. Finally, after the participants completed the questionnaire, we checked the questionnaire to ensure that there was no missing data. Then, we immediately collected, sealed, and encoded the questionnaire.

In the first survey, 490 employees' questionnaires were distributed, and 471 valid questionnaires were recovered. In the second survey, questionnaires were distributed to the employees who provided valid questionnaires for the first time and 457 valid ones were recovered. In the third survey, 443 questionnaires were distributed to the direct supervisors of employees who provided valid questionnaires for the second time, and the effective recovery rate was 90.41%. In terms of sample structure, most of the employees are male, accounting for 65.9% of the total. In terms of age structure, most of them are young people, and employees under the age of 35 account for 79.2%. In terms of education level, respondents with a bachelor's degree or beyond bachelor's degree account for 68.7% of the total.

### Measurements

The scales used in this study are mature ones used by many scholars at home and abroad. Each item adopts the Likert 6-point scale scoring method to measure five main variables: leader aggressive humor, bystander affective rumination, bystander workplace anxiety, bystander workplace withdrawal behavior, and bystander organization-based self-esteem.

For the measurement of leader aggressive humor, this study adopts the scale prepared by Martin et al., 2003, which has eight items in total. We revised the questionnaire according to the research situation, representative item is “If my colleague makes a mistake, my leader will dig at him/her”, and Cronbach's α is 0.76.

For the measurement of bystander affective rumination, this study adopts the affective rumination dimension scale in the three-dimensional degree of workplace rumination prepared by Cropley et al. (2012) to evaluate the degree of bystander affective rumination in the face of abusive management of colleagues, which has five items in total. The representative item is “After work, I feel nervous about thinking about work-related things”, and Cronbach's α is 0.70.

For the measurement of bystander workplace anxiety, this study adopts the scale developed by McCarthy and Goffin (2004), which has eight items in total. The representative item is “I feel nervous and worried about not meeting my performance goals”, and Cronbach's α is 0.83.

For the measurement of bystander workplace withdrawal behavior, this study adopts the scale developed by Lehman and Simpson (1992), which has 12 items in total. The representative item is “This employee is absent-minded at work”, and Cronbach's α is 0.77.

For the measurement of bystander organization-based self-esteem, this study uses the scale compiled by Pierce et al. (1989), which has ten items in total. The representative item is “I am valued in the organization”, and Cronbach's α is 0.74.

## Results

### Confirmatory factor analysis

In this study, Mplus7.4 is used for confirmatory factor analysis of related variables to test the discriminant validity between variables. Results as shown in Table 1, the five factor model has the best fitting effect (*x*^2^ = 256.98, *df* = 142, *x*^2^*/df* = 1.81, CFI = 0.95, TLI = 0.94, RMSEA = 0.04, SRMR = 0.05), indicating that the five variables in this study have good discriminant validity.

**Table 1:** Results of confirmatory factor analysis.

**Model**	**Factor**	***x* ^2^**	**df**	***x*^2^/df**	**CFI**	**TLI**	**RMSEA**	**SRMR**
Model 1	LAH + BAR + BWA + BWWB + BOBSE	1,258.75	152	8.28	0.53	0.47	0.13	0.11
Model 2	LAH + BAR + BWA + BWWB, BOBSE	1,121.41	151	7.43	0.59	0.53	0.12	0.10
Model 3	LAH + BAR + BWA, BWWB, BOBSE	769.01	149	5.16	0.74	0.70	0.10	0.08
Model 4	LAH + BAR, BWA, BWWB, BOBSE	560.77	146	3.84	0.82	0.79	0.08	0.08
Model 5	LAH, BAR, BWA, BWWB, BOBSE	256.98	142	1.81	0.95	0.94	0.04	0.05

*N* = 443; LAH, Leader Aggressive Humor; BAR, Bystander Affective Rumination; BWA, Bystander Workplace Anxiety; BWWB, Bystander Workplace Withdrawal Behavior; BOBSE, Bystander Organization-Based Self-Esteem; +Two factors combined as one.

### Correlation analysis

The mean value, SD, and correlation coefficient of each variable in this study are shown in Table 2. The data shows that the correlation between variables is consistent with the previous hypothesis of this study: Leader aggressive humor is significantly positively correlated with bystander affective rumination (γ = 0.19, *p* < 0.01), bystander workplace anxiety (γ = 0.31, *p* < 0.01), and bystander workplace withdrawal behavior (γ = 0.29, *p* < 0.01); Bystander affective rumination is significantly positively correlated with bystander workplace withdrawal behavior (γ = 0.38, *p* < 0.01); Bystander workplace anxiety is significantly positively correlated with bystander workplace withdrawal behavior (γ = 0.25, *p* < 0.01).

**Table 2:** Mean value, SD, and correlation coefficient of main variables.

	**Mean value (M)**	**Standard deviation (SD)**	**1**	**2**	**3**	**4**	**5**	**6**	**7**	**8**
1. Age	31.62	6.79	1							
2. Gender	0.34	0.48	−0.02	1						
3. Education level	2.20	0.53	0.02	−0.02	1					
4. Leader aggressive humor	4.87	0.78	0.05	−0.04	−0.11*	1				
5. Bystander affective rumination	5.46	0.57	0.12**	0.05	−0.12*	0.19**	1			
6. Bystander workplace anxiety	5.32	0.55	0.12**	−0.001	−0.02	0.31**	0.35**	1		
7. Bystander workplace withdrawal behavior	5.15	0.66	0.01	−0.01	−0.26**	0.29**	0.38**	0.25**	1	
8. Bystander organization-based self-esteem	5.41	0.48	−0.08	0.05	−0.09*	0.11*	0.18**	0.19**	0.42**	1

*N* = 443; **, *stand for *p* < 0.01, *p* < 0.05, respectively.

### Hypothesis testing

#### Main effects testing

**Figure 2:**
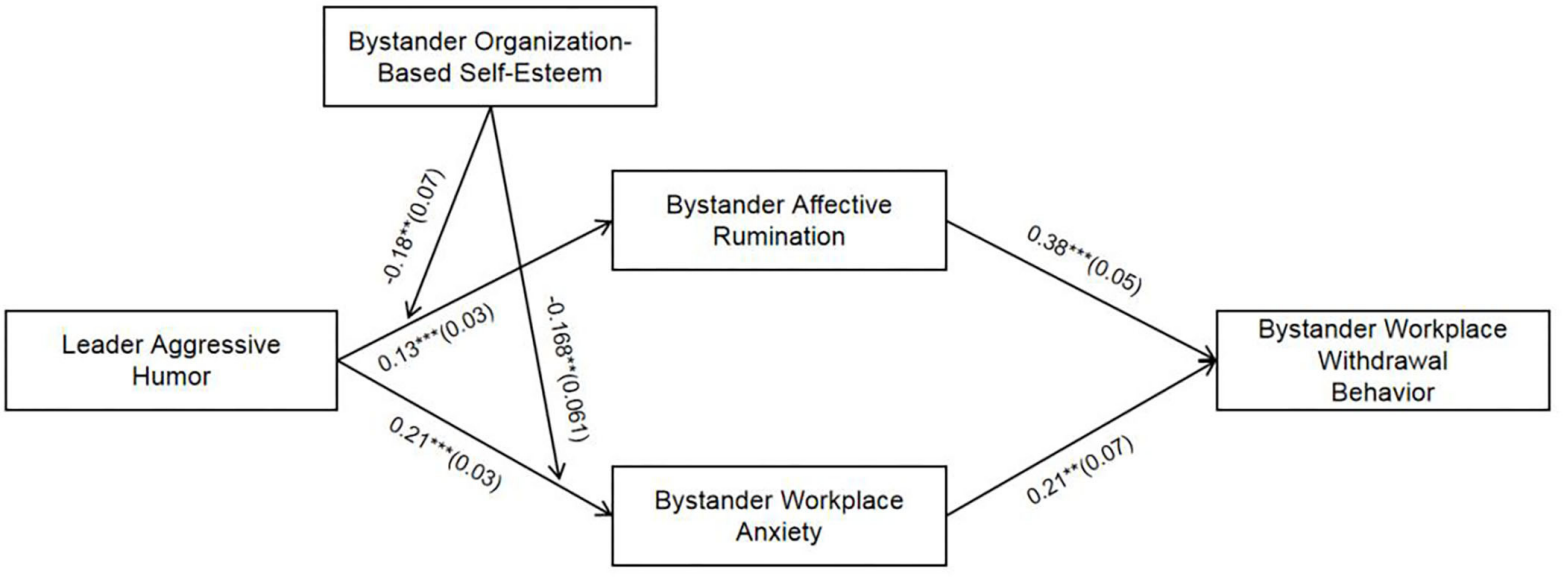
Path coefficient of the structural equation. *p < 0.05; **p < 0.01; ***p < 0.001; coefficients in the graph are standardized coefficients with standard errors in parentheses; control variables are age, gender, and education background.

Mplus 7.4 is used to test the fitting indexes and related hypotheses of the structural equation model. First, according to the fitting indexes of the theoretical model (*x*^2^ = 322.79, *df* = 145, *x*^2^/*df* = 2.23, CFI = 0.93, TLI = 0.91, RMSEA = 0.05, SRMR = 0.07), it can be judged that the fitting of the model is good. Second, the results of path analysis are shown in Figure 2. Leader aggressive humor is significantly positively correlated with bystander affective rumination (β = 0.13, *p* < 0.001) and bystander workplace anxiety (β = 0.21, *p* < 0.001), therefore, H1 and H2 are verified.

#### Mediating effects testing

This study uses bootstrap (repeated sampling 5,000 times) to test the mediation effect of bystander affective rumination and bystander workplace anxiety, respectively. The results are shown in Tables 3, 4. For mediation effect of bystander affective rumination (β = 0.05, *p* < 0.001), and the 95% confidence interval is (0.03, 0.09), excluding 0, therefore, H3 is verified. For mediation effect of bystander workplace anxiety (β = 0.05, *p* < 0.05), and the 95% CI is (0.02, 0.09), excluding 0, therefore, H4 is verified.

**Table 3:** Test results of mediation effect of bystander affective rumination.

**Model path**	**β**	***S.E*.**	***P***	**95% confidence interval**	
				**Lower**	**Upper**
Total effect	0.24	0.05	0.000	0.16	0.36
Direct effect	0.19	0.05	0.000	0.11	0.29
Indirect effect (LAH → BAR → BWWB)	0.05	0.02	0.000	0.03	0.09

*N* = 443; LAH, Leader Aggressive Humor; BAR, Bystander Affective Rumination; BWWB, Bystander Workplace Withdrawal Behavior; Bootstrap = 5,000 times.

**Table 4:** Test results of mediation effect of bystander workplace anxiety.

**Model path**	**β**	***S.E*.**	***P***	**95% confidence interval**	
				**Lower**	**Upper**
Total effect	0.24	0.05	0.000	0.16	0.36
Direct effect	0.20	0.05	0.000	0.12	0.30
Indirect effect (LAH → BWA → BWWB)	0.05	0.02	0.011	0.02	0.09

*N* = 443; LAH, Leader Aggressive Humor; BWA, Bystander Workplace Anxiety; BWWB, Bystander Workplace Withdrawal Behavior; Bootstrap = 5,000 times.

#### Moderating effect test

**Figure 3:**
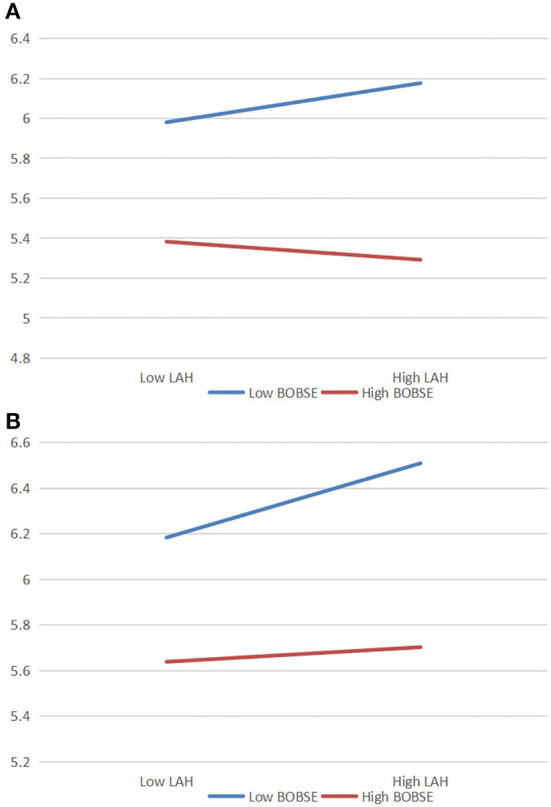
Moderation effect of bystander organization-based self-esteem between leader aggressive humor and bystander affective rumination **(A)**. Moderation effect of bystander organization-based self-esteem between leader aggressive humor and bystander workplace anxiety **(B)**. *N* = 443; LAH, leader aggressive humor; BOBSE, bystander organization-based self-esteem.

It can be seen from Figure 3 that the interaction between leader aggressive humor and bystander organization-based self-esteem has a significant effect on bystander affective rumination (β = −0.18, *p* < 0.01) and bystander workplace anxiety (β = −0.17, *p* < 0.01), indicating that bystander organization-based self-esteem significantly moderates the relationship between leader aggressive humor and bystander affective rumination, as well as the relationship between leader aggressive humor and bystander workplace anxiety. In order to further explain the moderation effect of bystander organization-based self-esteem, a simple slope test is carried out according to the suggestions of (Aiken and West, 1991) as shown in Figures 3A,B. When bystander organization-based self-esteem is low, leader aggressive humor has a strong positive effect on bystander affective rumination (β = 0.21, *t* = 4.59, *p* < 0.001) and bystander workplace anxiety (β = 0.29, *t* = 6.62, *p* < 0.001). When bystander organization-based self-esteem is high, leader aggressive humor has no significant positive effect on bystander affective rumination (β = 0.04, *t* = 0.82, *p* = 0.41), and has a weak positive effect on bystander workplace anxiety (β = 0.13, *t* = 2.96, *p* < 0.01). That is, the higher bystander organization-based self-esteem is, the weaker the positive effects of leader aggressive humor on both bystander affective rumination and bystander workplace anxiety are. Therefore, H5 and H6 are verified.

In order to test the moderation effect of bystander organization-based self-esteem, this study uses Latent Moderate Structural Equations (LMS) to test the moderated mediation effect (Fang and Weng, 2018). Results are shown in Tables 5A,B, the mediation effect of bystander affective rumination between leader aggressive humor and employee bystander workplace withdrawal behavior is moderated by bystander organization-based self-esteem. That is, for employees with high bystander organization-based self-esteem (one SD higher than the average), the indirect effect of leader aggressive humor on bystander workplace withdrawal behavior through bystander affective rumination is significantly lower than that of employees with low bystander organization-based self-esteem (one SD lower than the average), and the difference is significant (β = −0.07, *p* < 0.05), and 95% CI (−0.13, −0.02), excluding 0. Therefore, H7 is verified. The mediation effect of bystander workplace anxiety between leader aggressive humor and employee bystander workplace withdrawal behavior is moderated by bystander organization-based self-esteem. That is, for employees with high bystander organization-based self-esteem (one SD higher than the average value), the indirect effect of leader aggressive humor on bystander workplace withdrawal behavior through bystander workplace anxiety is significantly lower than that of employees with low bystander organization-based self-esteem (one SD lower than the average), and the difference is significant (β = −0.03, *p* < 0.05), and 95% confidence interval (−0.08, −0.01), excluding 0. Therefore, H8 is verified.

**Table 5:** Moderated mediation effect test results.

**Moderation variable**	**Path: LAH**→**BAR**→**BWWB**				
	**Indirect effect**	***S.E*.**	***P***	**95% confidence interval**	
				**Lower**	**Upper**
**(A)**			
Low BOBSE	0.08	0.02	0.000	0.05	0.13
High BOBSE	0.02	0.02	0.344	−0.02	0.05
Difference	−0.07	0.03	0.012	−0.13	−0.02
**(B)**			
Low BOBSE	0.06	0.02	0.009	0.02	0.11
High BOBSE	0.03	0.01	0.023	0.01	0.06
Difference	−0.03	0.02	0.047	−0.08	−0.01

*N* = 443; LAH, Leader Aggressive Humor; BWA, Bystander Workplace Anxiety; BWWB, Bystander Workplace Withdrawal Behavior; BOBSE, Bystander Organization-Based Self-Esteem; Bootstrap = 5,000 times.

## Discussion

### Theoretical implications

First, this study tests and verifies that leader aggressive humor plays a significant positive role on bystander workplace withdrawal behavior. For leader humor, the traditional research pays too much attention to constructive and positive exploration (Cooper and Sosik, 2012; Cooper et al., 2018), and pay insufficient attention to its negative effects, resulting in a deviation in the understanding of leader humor. Particularly, in the Chinese organizational context, the relationship between employees and leaders has the characteristics of formal hierarchical differences, power asymmetry, and inevitable social interaction. Therefore, from the perspective of bystanders, this study discusses leader aggressive humor and its dark side, constructs and verifies the mechanism model of leader aggressive humor on bystander workplace withdrawal behavior, and enriches and expands the research scope of leader humor effect.

Second, from the perspective of cognition-affection, this study verifies the mediation role of bystander affective rumination and bystander workplace anxiety between leader aggressive humor and bystander workplace withdrawal behavior. Previous studies have pointed out that high job requirements and negative workplace events are highly positively correlated with affective rumination and workplace anxiety (Hobman et al., 2009; Perko et al., 2017) and this study also verifies the same. When leaders show aggressive humor at work, bystanders show affective rumination and workplace anxiety through cognitive evaluation of negative information in the working environment. Besides, existing studies have confirmed that leadership behavior is an important antecedent of workplace withdrawal behavior (Walumbwa and Lawler, 2003; Tepper et al., 2009; Wei and Si, 2013), but few studies have explored it from the perspective of leader aggressive humor. This study broadens the research perspective of workplace withdrawal behavior to a certain extent and enriches the existing research results on workplace withdrawal behavior.

Finally, this study has introduced the moderation role of organization-based self-esteem between leader aggressive humor and bystander workplace withdrawal behavior. The results show that leader aggressive humor does not necessarily affect workplace withdrawal behaviors of all bystanders, while it is moderated by the level of bystander organization-based self-esteem. Leader aggressive humor has a great impact on workplace withdrawal behavior of bystanders with low organization-based self-esteem, but bystanders with high organization-based self-esteem are less affected by leader aggressive humor. Previous studies on organization-based self-esteem as a moderation variable mostly focused on positive situations (Pierce et al., 1993). This study expands the existing studies, discusses the regulation role of organization-based self-esteem in negative situations, and finds that high organization-based self-esteem can weaken the negative effect of leader aggressive humor on bystander workplace withdrawal behavior. It is verified that employees with high organization-based self-esteem do not care much about the influence of external environment and factors, and can focus more on the work itself.

### Practical implications

First, organizations should pay attention to and be alert to the negative effects brought by leader aggressive humor. Leader humor can help people relieve tension and improve the organizational atmosphere, leader aggressive humor may bring more negative effects, which will not only hurt colleagues who are ridiculed, but also have a negative impact on bystanders. Therefore, organizations should prevent and reduce the occurrence of leader aggressive humor. In this sense, organizations can carry out targeted leadership training, improve leaders' personal cultivation at work, implement humanized management, promote leaders to truly realize the possible adverse effects of aggressive humor, and correct leaders' aggressive humor behavior from the origin.

Second, organizations should focus on affective changes in employees. On the one hand, when guiding employees' work, leaders should minimize negative words and avoid affective rumination. Besides, employees with great affective changes after work should be given psychological counseling and encouragement in time to reduce or even eliminate the negative impact of affective rumination. On the other hand, organizations should design corresponding training courses (such as skill improvement and psychological quality training) to improve employees' cognitive and affective regulation ability, so that employees can work in a more ideal affective state. At the same time, it is also necessary to provide corresponding employee assistance bases (such as a mental health room and fitness room), and regularly hold corresponding cultural and recreational activities as well as competitions to intervene in the possible negative psychology of employees and improve the coping ability of negative affections. In addition, it is more critical to establish a positive and open organizational culture and atmosphere, which can improve employees' psychological state more effectively (Cheng and Mccarthy, 2018).

Finally, organizations should regularly monitor the level of employees' organization-based self-esteem and formulate personalized management policies. For employees with low levels, organizations should respect their subject status and value, and encourage them to actively participate in decision-making by implementing positive incentives. Also, organizations should care more about employees, making them realize that they are important employees for the organizations. In these ways, organizations can promote them to produce higher organization-based self-esteem and stimulate their work enthusiasm. For employees with high levels of organization-based self-esteem, organizations should give them a certain amount of work autonomy and certain resources to provide opportunities and guarantees for promoting organizational performance. Moreover, organizations can adopt certain methods to select employees with high organization-based self-esteem and also adopt certain training to improve employees' organization-based self-esteem.

## Limitations and directions for future research

This study also has some limitations, which need to be further explored in the future. First, the survey objects of this study come from all walks of the industry. Employees in different industries and different types of employees (such as knowledge workers and manual workers) may have different ways of leader aggressive humor and bystander workplace withdrawal behavior. Therefore, the conclusions of this study can be further verified among employees in a certain industry or a certain type of employee in the future. Second, the importance of content factors of leader aggressive humor is ignored in measurement, such as non-verbal factors (leader facial expression). In the future, the impact of content-based leader aggressive humor on employees' attitudes and behavior can be investigated. Third, this study mainly discusses the results of leader aggressive humor from the individual level. However, leader aggressive humor may also affect the team, organization, and other levels. For example, group ridiculing (despising and ridiculing many people) may cause dissatisfaction among many people and disharmony within the team. Therefore, future research can deeply explore the causes and consequences of leader aggressive humor from the levels of group, team, and organization. Based on different theories, more research on leader aggressive humor from different angles can be carried out in the future. Fourth, due to our limited social resources, the samples of this study mainly focus on the data survey results in some parts of China. There may be some defects in the external validity of the sample categories, and the universality of the research results needs to be further confirmed. In the future, researchers can expand the sample range or conduct cross-cultural research, and obtain samples in different countries and regions, so that the results can be more convincing.

## Conclusion

From the perspective of bystanders, this study intends to explore the influence mechanism and boundary conditions of leader aggressive humor on bystander workplace withdrawal behavior based on the dual path of cognition and affection. The survey data verifies that both bystander affective rumination and bystander workplace anxiety play a mediation role between leader aggressive humor and bystander workplace withdrawal behavior. Bystander organization-based self-esteem plays a moderation role. Leader aggressive humor can cause bystander affective rumination and bystander workplace anxiety which leads to bystander workplace withdrawal. Bystander organization-based self-esteem effectively weakens the positive effect of leader aggressive humor on bystander affective rumination and bystander workplace anxiety, as well as the mediation effect of bystander effective rumination and bystander workplace anxiety.

## Data availability statement

The raw data supporting the conclusions of this article will be made available by the authors, without undue reservation.

## Ethics statement

Ethical review and approval was not required for the study on human participants in accordance with the local legislation and institutional requirements. Written informed consent from the patients/ participants or patients/participants legal guardian/next of kin was not required to participate in this study in accordance with the national legislation and the institutional requirements.

## Author contributions

HC and JB: conceptualization. HC and LW: methodology and validation. LW: software and investigation. HC: formal analysis and data curation. JB: resources. HC, LW, and JB: writing—original draft preparation. All the authors contributed to the article and approved the submitted version.

## Conflict of interest

The authors declare that the research was conducted in the absence of any commercial or financial relationships that could be construed as a potential conflict of interest.

## Publisher's note

All claims expressed in this article are solely those of the authors and do not necessarily represent those of their affiliated organizations, or those of the publisher, the editors and the reviewers. Any product that may be evaluated in this article, or claim that may be made by its manufacturer, is not guaranteed or endorsed by the publisher.

## References

[B1] Aiken L. S. West S. G. (1991). Multiple Regression: Testing and Interpreting Interactions. Thousand Oaks, CA: Sage.

[B2] Anderson W. Ditunnariello N. (2016). Aggressive humor as a negative relational maintenance behavior during times of conflict. Qual. Rep. 21, 1513–1530. 10.46743/2160-3715/2016.2149

[B3] Bamberger P. A. Bacharach S. B. (2006). Abusive supervision and subordinate problem drinking: Taking resistance, stress and subordinate personality into account. Hum. Relat. 59, 723–752. 10.1177/0018726706066852

[B4] Barlow D. H. (2004). Anxiety and Its Disorders: The Nature and Treatment of Anxiety and Panic. New York: Guilford Press.

[B5] Beckker H. L. Legare F. Stacey D. O'Connor A. Lemyre L. (2003). Anxiety a suitable measure of decision aid effectiveness: a systematic review? Pat. Educ. Counsel. 50, 255–262. 10.1016/S0738-3991(03)00045-4 12900095

[B6] Beerhr T. A. (1995). Psychological Stress in the Workplace. London: Routledge.

[B7] Berset M. Elfering A. Lüthy S. Lüthi S. Semmer N. K. (2011). Work stressors and impaired sleep: rumination as a mediator. Stress Health 27, 71–82. 10.1002/smi.1337 27486625

[B8] Bowling N. A. Eschleman K. J. Wang Q Kirkendall C Alarcon G. (2010). A meta-analysis of the predictors and consequences of organization-based self-esteem. J. Occup. Organ. Psychol. 83, 601–626. 10.1348/096317909X454382

[B9] Chen Z. X. Aryee S. (2007). Delegation and employee work outcomes:an examination of the cultural context of mediation processes in China. Acad. Manag. J. 50, 226–238. 10.5465/amj.2007.24162389

[B10] Cheng B. H. Mccarthy J. M. (2018). Understanding the dark and bright sides of anxiety: a theory of workplace anxiety. J. Appl. Psychol. 103, 537–560. 10.1037/apl0000266 29355338

[B11] Chi S. C. S. Liang S. G. (2013). When do subordinates' emotion-regulation strategies matter? Abusive supervision, subordinates' emotional exhaustion, and work withdrawal. Leadership Q. 24, 125–137. 10.1016/j.leaqua.2012.08.006

[B12] Cooper C. (2008). Elucidating the bonds of workplace humor: a relational process model. Hum. Relat. 61, 1087–1115. 10.1177/0018726708094861

[B13] Cooper C. D. Kong D. T. Crossley C. D. (2018). Leader humor as an interpersonal resource: integrating three theoretical perspectives. Acad. Manag. J. 61, 769–796. 10.5465/amj.2014.0358

[B14] Cooper C. D. Sosik J. J. (2012). “The laughter advantage: cultivating high-quality connections and workplace outcomes through humor,” in The Oxford Handbook of Positive Organizational Scholarship, eds K. S. Cameron and G. M. Spreitzer (New York, NY: Oxford University Press), 474–489.

[B15] Cropanzano R. Wright T. A. (2001). When a “happy” worker is really a “productive” worker: a review and further refifinement of the happy-productive worker thesis. Consult. Psychol. J. Pract. Res. 53, 182–199. 10.1037/1061-4087.53.3.18231861812

[B16] Cropley M. Michalianou G. Pravettoni G. Millward L. J. (2012). The relation of post-work ruminative thinking with eating behaviour. Stress Health 28, 23–30. 10.1002/smi.1397 22259155

[B17] Fang J. Weng Z. L. (2018). The analyses of moderated mediation effects based on structural equation modeling. J. Psychol. Sci. 41, 453–458. 10.16719/j.cnki.1671-6981.20180231

[B18] Forgas J. P. George J. M. (2001). Affective influences on judgments and behavior in organizations: an information processing perspective. Organ. Behav. Hum. Decis. Process. 86, 3–34. 10.1006/obhd.2001.2971

[B19] Goswami A. Nair P. K. Grossenbacher M. A. (2015). Impact of aggressive humor on dysfunctional resistance. Pers. Individ. Dif. 74, 265–269. 10.1016/j.paid.2014.10.037

[B20] Haines J. Williams C. L. Carson J. M. (2002). Workplace phobia: psychological and psychophysiological mechanisms. Int. J. Stress Manag. 9, 129–145. 10.1023/A:1015518030340

[B21] Hobfoll S. E. Halbesleben J. Neveu J. P. Westman M. (2018). Conservation of resources in the organizational context: the reality of resources and their consequences. Annu. Rev. Organ. Psychol. Organ. Behav. 5, 103–128. 10.1146/annurev-orgpsych-032117-104640

[B22] Hobman E. V. Restubog S. L. D. Bordia P. Tang R. L. (2009). Abusive supervision in advising relationships: investigating the role of social support. Appl. Psychol. 58, 233–256. 10.1111/j.1464-0597.2008.00330.x

[B23] Huo Y. Lam W. Chen Z. (2012). Am i the only one this supervisor is laughing at? Effects of aggressive humor on employee strain and addictive behaviors. Person. Psychol. 65, 859–885. 10.1111/peps.12004

[B24] Jones M. K. Latreille P. L. Sloane P. J. (2015). Job anxiety, work-related psychological iiness and workplace performance. Br. J. Ind. Relat. 54, 742–767. 10.1111/bjir.12159

[B25] Lehman W. E. Simpson D. D. (1992). Employee substance use and on-the-job behaviors. J. Appl. Psychol. 77, 309–321. 10.1037/0021-9010.77.3.309 1601823

[B26] March J. G. Simon H. A. (1958). Organizations. New York: Wiley.

[B27] Martin R. A. Puhlik-Doris P. Larsen G. Gray J. Weir K. (2003). Individual differences in uses of humor and their relation to psychological well-being: development of the humor styles questionnaire. J. Res. Pers. 37, 48–75. 10.1016/S0092-6566(02)00534-2

[B28] McCarthy J. Goffin R. (2004). Measuring job interview anxiety: beyond weak knees and sweaty palms. Pers. Psychol. 57, 607–637. 10.1111/j.1744-6570.2004.00002.x

[B29] McCarthy J. M. Trougakos J. P. Cheng B. H. (2016). Are anxious workers less productive workers? It depends on the quality of social exchange. J. Appl. Psychol. 101, 279–291. 10.1037/apl0000044 26375962

[B30] Mischel W. Shoda Y. (1995). A cognitive-affective system theory of personality: reconceptualizing situations, dispositions, dynamics, and invariance in personality structure. Psychol. Rev. 102, 246–268. 10.1037/0033-295X.102.2.246 7740090

[B31] Mitchell M. S. Vogel R. M. Folger R. (2012). “Beyond the consequences to the victim: The impact of abusive supervision on third party observers,” in Handbook of Unethical Work Behavior: Implications for Well-being, eds R. A. Giacalone, and M. D. Promislo (Armonk, NY: M.E. Sharpe), 21–43.

[B32] Mitchell M. S. Vogel R. M. Folger R. (2015). Third parties'reactions to the abusive supervision of coworkers. J. Appl. Psychol. 100, 1040–1055. 10.1037/apl0000002 25243999

[B33] Morrison E. W. Robinson S. L. (1997). When employees feel betrayed: a model of how psychological contract violation develops. Acad. Manag. Rev. 2, 226–256. 10.2307/259230

[B34] Perko K. Kinnunen U. Feldt T. (2017). Long-term profiles of work-related rumination associated with leadership, job demands,and exhaustion: a three-wave study. Work Stress 31, 395–420. 10.1080/02678373.2017.1330835

[B35] Pierce J. L. Garden D. G. (2004). Self-esteem within the work and organizational context: a review of the organization-based self-esteem literature. J. Manage. 30, 591–622. 10.1016/j.jm.2003.10.001

[B36] Pierce J. L. Gardner D. G. Crowley C. (2016). Organization-based self-esteem and well-being: empirical examination of a spillover effect. Eur. J. Work Org. Psychol. 25, 181–199. 10.1080/1359432X.2015.1028377

[B37] Pierce J. L. Gardner D. G. Cummings L. L. (1989). Organization-based self-esteem: construct definition, measurement, and validation. Acad. Manag. J. 32, 622–648. 10.5465/256437

[B38] Pierce J. L. Gardner D. G. Dunham R. B. Cummings L. L. (1993). Moderation by organization-based self-esteem of role condition-employee response relationships. Acad. Manag. J. 36, 271–288. 10.5465/256523

[B39] Podsakoff N. P. LePine J. A. LePine M. A. (2007). Differential challenge stressor-hindrance stressor relationships with job attitudes, turnover intentions, turnover, and withdrawal behavior: a meta-analysis. J. Appl. Psychol. 92, 438–454. 10.1037/0021-9010.92.2.438 17371090

[B40] Priesemuth M. Schminke M. (2019). Helping thy neighbor? Prosocial reactions to observed abusive supervision in the workplace. J. Manag. 45, 1225–1251. 10.1177/0149206317702219

[B41] Priesemuth M. Schminke M. Ambrose M. L. Folger R. (2014). Abusive supervision climate: a multiple-mediation model of its impact on group outcomes. Acad. Manag. J. 57, 1513–1534. 10.5465/amj.2011.0237

[B42] Probst T. M. (2002). “The impact of job insecurity on employee work attitudes,job adaptation,and organizational withdrawal behaviours,” in The Psychology of Work: Theoretically Based Empirical Research, eds J. M. Brett, and F. Drasgow (Mahwah, NJ: Lawrence Erlbaum Associates), 141–168.

[B43] Pundt A. Herrmann F. (2015). Affiliative and aggressive humour in leadership and their relationship to leader-member exchange. J. Occup. Organ. Psychol. 88, 108–125. 10.1111/joop.12081

[B44] Pundt A. Venz L. (2017). Personal need for structure as a boundary condition for humor in leadership. J. Organ. Behav. 38, 87–107. 10.1002/job.2112

[B45] Querstret D. Cropley M. (2012). Exploring the relationship between work-related rumination, sleep quality, and work-related fatigue. J. Occup. Health Psychol. 17, 341–353. 10.1037/a0028552 22746369

[B46] Robert C. Dunne T. C. Iun J. (2016). The impact of leader humor on subordinate job satisfaction: the crucial role of leader–subordinate relationship quality. Group Org. Manag. 41, 375–406. 10.1177/1059601115598719

[B47] Rosenberg C. Walker A. Leiter M. Graffam J. (2021). Humor in workplace leadership: a systematic search scoping review. Front. Psychol. 12, 610795. 10.3389/fpsyg.2021.610795 34385944 PMC8353333

[B48] Syrek C. J. Weigelt O. Peifer C. Antoni C. H. (2017). Zeigarnik's sleepless nights: how unfifinished tasks at the end of the week impair employee sleep on the weekend through rumination. J. Occup. Health Psychol. 22, 225–238. 10.1037/ocp0000031 27101340

[B49] Tepper B. J. Carr J. C. Breaux D. M. Geider S. Hu C. Hua W. (2009). Abusive supervision, intentions to quit, and employees' workplace deviance: a power/dependence analysis. Org. Behav. Hum. Decis. Process. 109, 156–167. 10.1016/j.obhdp.2009.03.004

[B50] Tepper B. J. Henle C. Lambert L. S. Giacalone R. (2008). Abusive supervision and subordinates'organizational deviance. J. Appl. Psychol. 93, 721–732. 10.1037/0021-9010.93.4.721 18642979

[B51] Vecchio R. P. Justin J. E. Pearce C. L. (2009). The Inflfluence of Leader Humor on relationships between leader behavior and follower outcomes. J. Manag. Issues 21, 171–194.

[B52] Viswesvaran C. (2002). Absenteeism and measures of job performance: a meta analysis. Int. J. Select. Assess. 10–2, 12–17. 10.1111/1468-2389.00190

[B53] Walumbwa F. O. Lawler J. J. (2003). Building effective organizations:transformational leadership, collectivist orientation, work-related attitudes and withdrawal behaviours in three emerging economies. Int. J. Hum. Resour. Manag. 14, 1083–1101. 10.1080/0958519032000114219

[B54] Wang S. Yi X. (2012). Organizational justice and work withdrawal in Chinese companies: the moderating effects of allocentrism and idiocentrism. Int. J. Cross Cult. Manag. 12, 211–228. 10.1177/1470595812439871

[B55] Wei F. Si S. (2013). Tit for tat? Abusive supervision and counterproductive work behaviors: the moderating effects of locus of control and perceived mobility. Asia Pac. J. Manag. 30, 281–296. 10.1007/s10490-011-9251-y

[B56] Wisse B. Rietzschel E. (2014). Humor in leader-follower relationships: humor styles, similarity and relationship quality. Humor 27, 249–269. 10.1515/humor-2014-0017

[B57] Yam K. C. Christian M. S. Wei W. Liao Z. Nai J. (2018). The mixed blessing of leader sense of humor: examining costs and benefifits. Acad. Manag. J. 61, 348–369. 10.5465/amj.2015.1088

[B58] Yin K. Liu Y. R. Liu M. (2014). Organization-based self-esteem: a review. Hum. Resour. Dev. China 28, 38–47. 10.16471/j.cnki.11-2822/c.2014.05.01834408692

[B59] Zimmerman R. D. Darnold T. C. (2009). The impact of job performance on employee turnover intentions and the voluntary turnover process: a meta-analysis and path model. Person. Rev. 38, 142–158. 10.1108/00483480910931316

